# Report of a Surgical Case

**Published:** 1901-10

**Authors:** J. S. Greenleaf

**Affiliations:** Assistant Surgeon Seaboard Air Line Railway, Ulmus, S. C.


### REPORT OF A SURGICAL CASE.

BY J. S. GREENLEAF, M.D.,

Assistant Surgeon Seaboard Air Line Railway, Ulmus, S. C.

After reviewing the literature and text-books on this subject, I was unable to find a similar case reported.

The patient was brought to my office on August 29th, and gave the following history:

He said that he was working at a sawmill and a piece of timber was overbalanced on the roll-way, and one end struck him under the chin, causing a lacerated wound of 1¼ inches, tearing away the larger part of the flesh from the inferior maxillary bone, which caused quite a severe hemorrhage. This accident occurred late in the evening of August 28th, and the foreman of the mill sent him to my office on the 29th of August for treatment.

*Treatment*: The wound was greatly swollen, but I managed to wash it out thoroughly with hydrogen peroxide and afterwards with bichloride of mercury solution 1-2000, and then with a warm sterilized salt solution, and took three stitches in the wound with a silk thread, then packed the wound with iodoform gauze, 10 per cent., and ordered the patient to report to my office again on August 30th, which he did. After examining the wound I found no sign of any septic infection; I ordered him to return on August 31st to have his wound redressed, and I then found the wound in a healthy condition, and I ordered him to return on September 3d, when I removed the stitches and found the wound granulating from the bottom. I again dressed the wound with iodoform gauze 10 per cent. and the patient returned to work on September 5th, and I never saw him any more, but I was informed by the foreman of the mill that he had been working steadily every day since September 5th, and his wound was nearly entirely healed, leaving only a scar.

